# An Underactuated Hip Exoskeleton to Assist Hip Joints Driven by a Single-Series Elastic Actuator

**DOI:** 10.3390/biomimetics11080561

**Published:** 2026-08-06

**Authors:** Yangshuo Yue, Weijie Zhao, Jiaxu Wang, Zelin Yu, Zhiheng Zha, Bai Chen, Shengli Chen, Xiaoang Xu

**Affiliations:** 1 Jiangsu Product Quality Testing and Inspection Institute, Nanjing 210007, China; yue.yangshuo@jszj.net.cn (Y.Y.); chenshl@mail.ustc.edu.cn (S.C.); xuxa@jszj.net.cn (X.X.); 2 College of Mechanical and Electrical Engineering, Nanjing University of Aeronautics and Astronautics, Nanjing 210016, China; wangjiaxu2024@nuaa.edu.cn (J.W.); yuzelin@nuaa.edu.cn (Z.Y.); zhazhiheng@nuaa.edu.cn (Z.Z.); chenbye@nuaa.edu.cn (B.C.)

**Keywords:** hip exoskeleton, underactuated mechanism, series elastic actuator, walking assistance

## Abstract

Conventional hip exoskeletons typically employ multiple actuators to provide effective assistance to the corresponding joint, leading to an increase in the weight of the exoskeleton. Underactuated designs reduce the number of actuators, thereby lowering system weight and cost. However, existing single-motor underactuated hip exoskeletons still face challenges in achieving precise assistance and accommodating non-walking movements such as free sitting. In this work, we propose an underactuated hip exoskeleton with a series elastic actuator (SEA) and two independent cables for walking assistance. The incorporation of the SEA contributes to system safety and precise assistive force control. Furthermore, the proposed differential cable structure enables free sitting movement and allows for non-strictly symmetric hip motion. In experiments, with a target assistive force of 300 N, the proposed actuator achieves a peak force-tracking accuracy of 98.01% in walking tests, and the hip exoskeleton reduces peak muscle activation by up to 23.62% during walking.

## 1. Introduction

Walking is one of the most frequently performed forms of human locomotion in daily life. During walking, the hip joint plays a critical role, providing substantial torque support for gait [1]. With the acceleration of population aging and the growing interest of younger generations in outdoor activities, the demand for walking-assistive devices has steadily increased in modern society. In recent years, walking assistive devices have been extensively studied. During walking, the hip joint accounts for a large proportion of the exerted force [2]; therefore, a hip exoskeleton is an effective walking assistance device that can be worn by users to provide assistive torque at the hip joint, thereby reducing the mechanical load during walking and lowering metabolic cost [3].

As a wearable walking-assistive system, a hip exoskeleton must provide assistive torques with appropriate magnitude and precise timing during walking to effectively reduce human metabolic energy expenditure [3,4]. Passive exoskeletons are advantageous in terms of lightweight design and low cost. However, their assistive torque is constrained by the inherent properties of elastic elements, and the lack of active control limits precise regulation of assistance magnitude and timing [5]. Active hip exoskeleton systems typically employ two drive motors to deliver assistive torques independently to the left and right hip joints. The hip exoskeleton devices proposed in [6,7,8,9] utilize two actuators to achieve active assistance of the corresponding bilateral hip joint. However, due to the use of two actuators, the control complexity of such active hip exoskeletons is high, joint coordination is relatively low, and the device weight is consequently increased. Consequently, novel hip exoskeletons employing underactuated structures to reduce the number of actuators have developed rapidly in recent years.

Underactuated exoskeletons reduce the number of actuators, which can effectively lower control complexity while also helping to reduce device weight and manufacturing costs. For hip exoskeletons, the design should adhere to the fundamental principles of human gait. During normal walking, the angular position of the hip joint follows a periodic trajectory that can be approximated by a sinusoidal waveform, similar to that of an undamped oscillator [10]. To ensure walking comfort and daily usability, the design should accommodate non-strictly symmetric leg motion during walking and allow for free sitting movement. Meanwhile, while reducing the number of actuators, it is necessary to achieve effective force-control performance in order to provide users with accurate torque assistance [11,12].

However, current underactuated hip exoskeleton devices are unable to fully meet all of the aforementioned requirements. Reference [13] proposed a hip-assist exoskeleton based on an underactuated soft design in which a single actuator drives bilateral hip flexion, effectively reducing system complexity and enabling a lightweight structure. Reference [14] proposed an underactuated modular exoskeleton system in which the hip module employs a single motor combined with Bowden cable transmission to provide assistance to both lower limbs. However, these devices employ Bowden cables for force transmission, which results in low force-control accuracy. Moreover, they mainly focus on walking assistance, while adaptations to non-walking movements (e.g., free sitting movement) have not yet been fully discussed.

To overcome the above challenges, we design a novel underactuated hip exoskeleton in which a single actuator provides assistance to both hip joints. In the actuator, a series elastic actuator (SEA) is introduced to enhance system compliance, allowing for precise force control while improving overall safety. Two independent cables are employed to connect the two sides of the actuator to the left and right hip joints, forming a differential cable structure. The resulting differential cable structure allows for non-strictly symmetric leg motion, reducing kinematic constraints on the wearer’s natural hip movement. Meanwhile, this structure allows users to perform natural movements, such as sitting down. During assisted walking experiments, the hip exoskeleton provided assistance to both hip joints throughout the entire gait cycle, with a peak force-tracking accuracy of 98.01% in walking tests and hip exoskeleton reducing peak muscle activation by up to 23.62% during walking.

The main contributions of the proposed mechanism can be summarized as follows:

1. The underactuated mechanism reduces the number of actuators, effectively decreasing control complexity while also lowering the device weight.
2. The differential cable structure enables free sitting movement by allowing the drive disks to rotate in the same direction without actuating the SEA.
3. During walking, the same differential structure allows for non-strictly symmetric forward and backward hip motion, thereby reducing kinematic constraints on the wearer and helping improve motion comfort.

The remainder of this paper is organized as follows. Section 2 presents the design of the hip exoskeleton. Section 3 provides a model of the SEA and describes the assistive strategies employed in this study. Section 4 details several experiments conducted to validate the structure of the proposed hip exoskeleton and assistive strategies. Section 5 and Section 6 discuss and conclude this work.

## 2. Hip Exoskeleton Design

### 2.1. Working Principle of a Hip Exoskeleton

The purpose of a hip exoskeleton is to provide appropriate assistive torque during walking in accordance with the hip-joint force-generation patterns, thereby reducing the user’s metabolic energy expenditure. The human hip joint has three degrees of freedom, and during normal walking, the sagittal-plane degree of freedom bears the majority of the joint torque [2].

**Figure 1:**
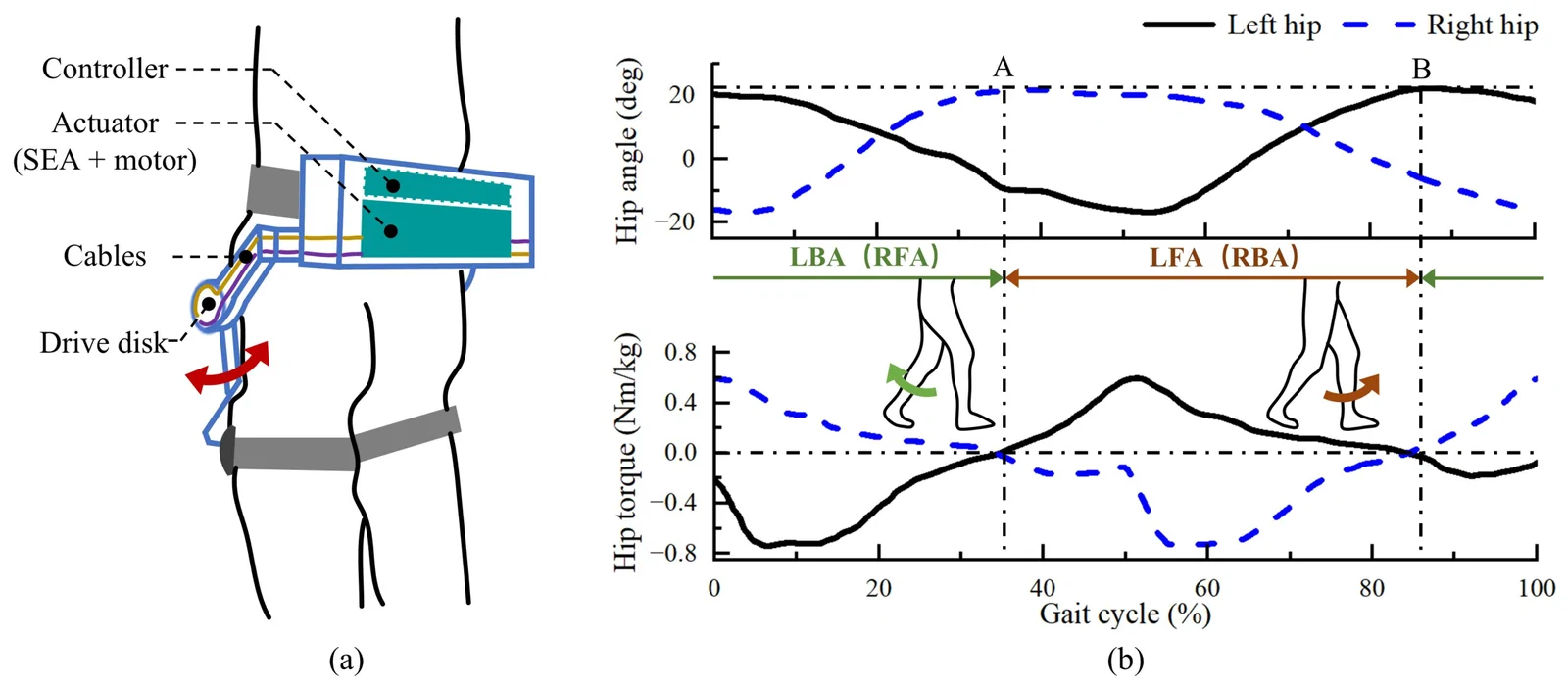
(**a**) Basic design of the hip exoskeleton. (**b**) Gait-cycle analysis of a human hip joint. Points A and B represent the moments of maximum right and left hip angles, respectively, within a gait cycle.

Therefore, we designed a hip exoskeleton featuring a single actuator coupled with both hip joints to provide force assistance during normal walking, as shown in Figure 1a, thereby minimizing the burden associated with gait. The actuator is positioned at the waist and incorporates a drive motor, an SEA, and related electronic components. Assistive forces are delivered to the drive disks through cable transmission, and the underactuated mechanism applies assistive force to the two legs in opposite directions to facilitate walking assistance.

The principle of the hip exoskeleton is based on a design derived from the gait cycle. Figure 1b illustrates the hip-joint angle and torque profiles over a walking-gait cycle [2,15], with the gait cycle beginning with a left heel strike. Throughout the complete gait cycle, the hip-joint angle and moment curves exhibit a sinusoidal pattern; this conclusion is discussed in detail in Reference [10]. We divide the aforementioned gait cycle into two stages:

Stage-1: From point A to point B:
  During this stage, the right hip-joint angle gradually decreases from its maximum value, and the joint torque is negative; meanwhile, the left hip-joint angle roughly shows an increasing trend, and the joint torque is positive. Therefore, forward assistance is applied to the left hip to help with walking (left forward assistance, LFA); due to the mechanical design of the mechanism, this assistance manifests simultaneously as backward assistance at the right hip (right backward assistance, RBA).
Stage-2: From point B to point A of the next gait cycle:
  In this stage, the left hip-joint angle gradually decreases from its maximum value, and the joint torque is negative, while the right hip-joint angle generally shows an increasing trend and the joint torque is positive. The system applies backward assistance to the left hip to assist in walking (left backward assistance, LBA); correspondingly, the right hip receives forward assistance (rRight forward assistance, RFA).

Based on the above division of gait-cycle phases, a support strategy targeting the force characteristics of the two phases is designed later for walking assistance.

### 2.2. Working Principle of the Actuator and Mechanical Structures

**Figure 2:**
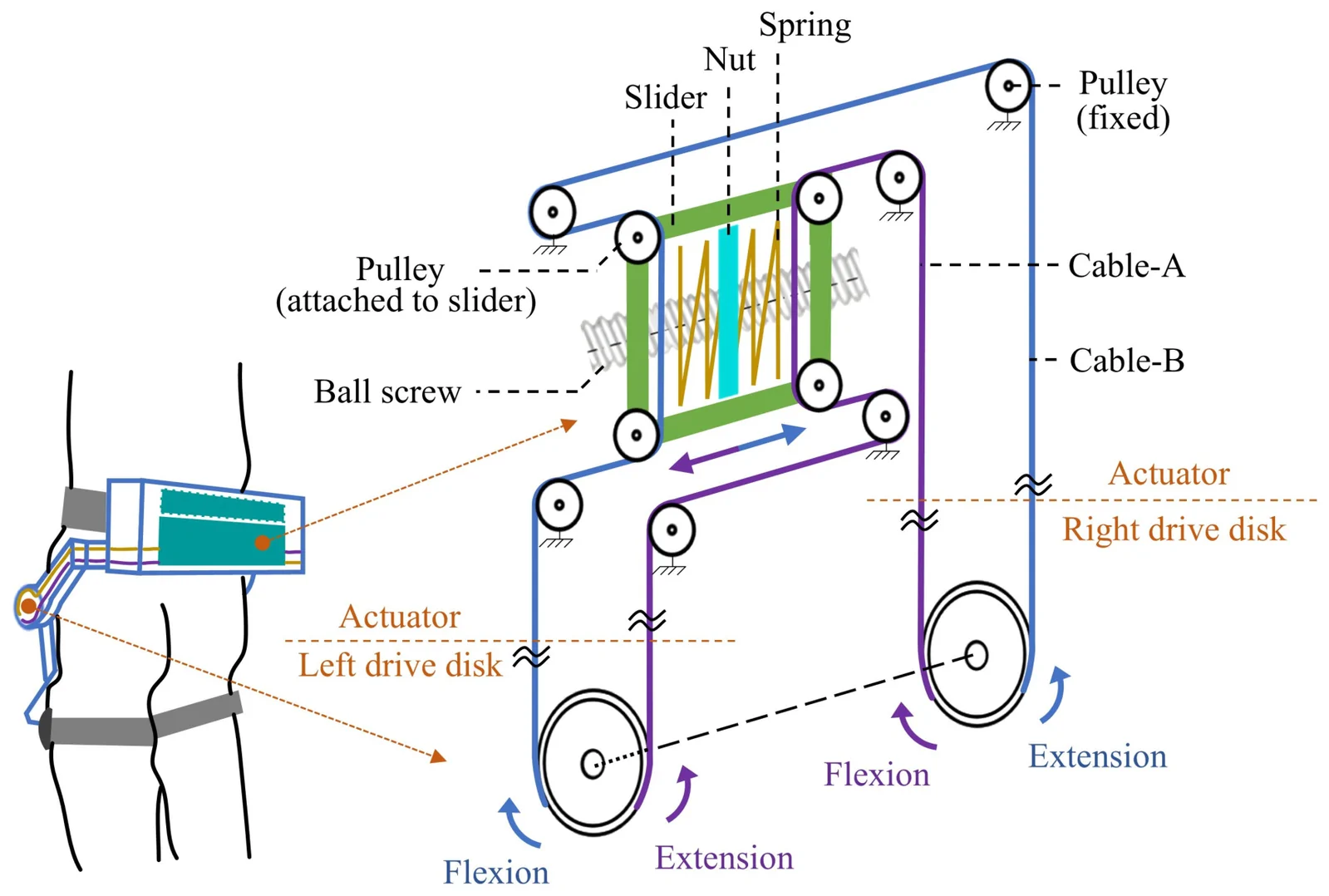
Working principle of the actuator.

The actuator mainly consists of a motor and an SEA, and its working principle is illustrated in the Figure 2. The SEA is primarily composed of a ball screw, a slider, springs, cables, and pulleys. The slider is mounted on a linear guide rail and is able to move along the ball screw. The springs are installed between the slider and the ball-screw nut. Two identical springs are selected to ensure symmetric assistance to the left and right sides during walking. Pulleys are mounted on both sides of the slider to provide bilateral driving forces, while the remaining pulleys are fixed on the main frame of the actuator.

The operating principle of the underactuated mechanism is illustrated as follows. During assistance, the motor drives the lead screw to rotate, causing the slider to translate axially toward one side. The tension in the cable on that side increases, thereby driving the left and right hip-joint drive disks to rotate in opposite directions. When the slider translates toward the opposite side, the other cable is tensioned, resulting in a reversal of the rotation directions of both hip-joint drive disks. Through this alternating motion, gait assistance is realized via the underactuated transmission structure.

The working principle of the differential cable structure is illustrated as follows. The drive system incorporates two independent actuation cables that are fixed to opposite sides of the slider and connected to different ends of the hip-joint drive disks, thereby enabling assistive torque generation during walking. When the slider remains stationary, the mechanical configuration allows the left and right drive disks to rotate in the same direction, permitting free sitting movement. Based on the above principles, the proposed differential structure allows for non-strictly symmetric forward and backward hip motion, which can reduce kinematic constraints on the wearer and help improve motion comfort while walking.

### 2.3. Prototype Design of the Hip Exoskeleton

**Figure 3:**
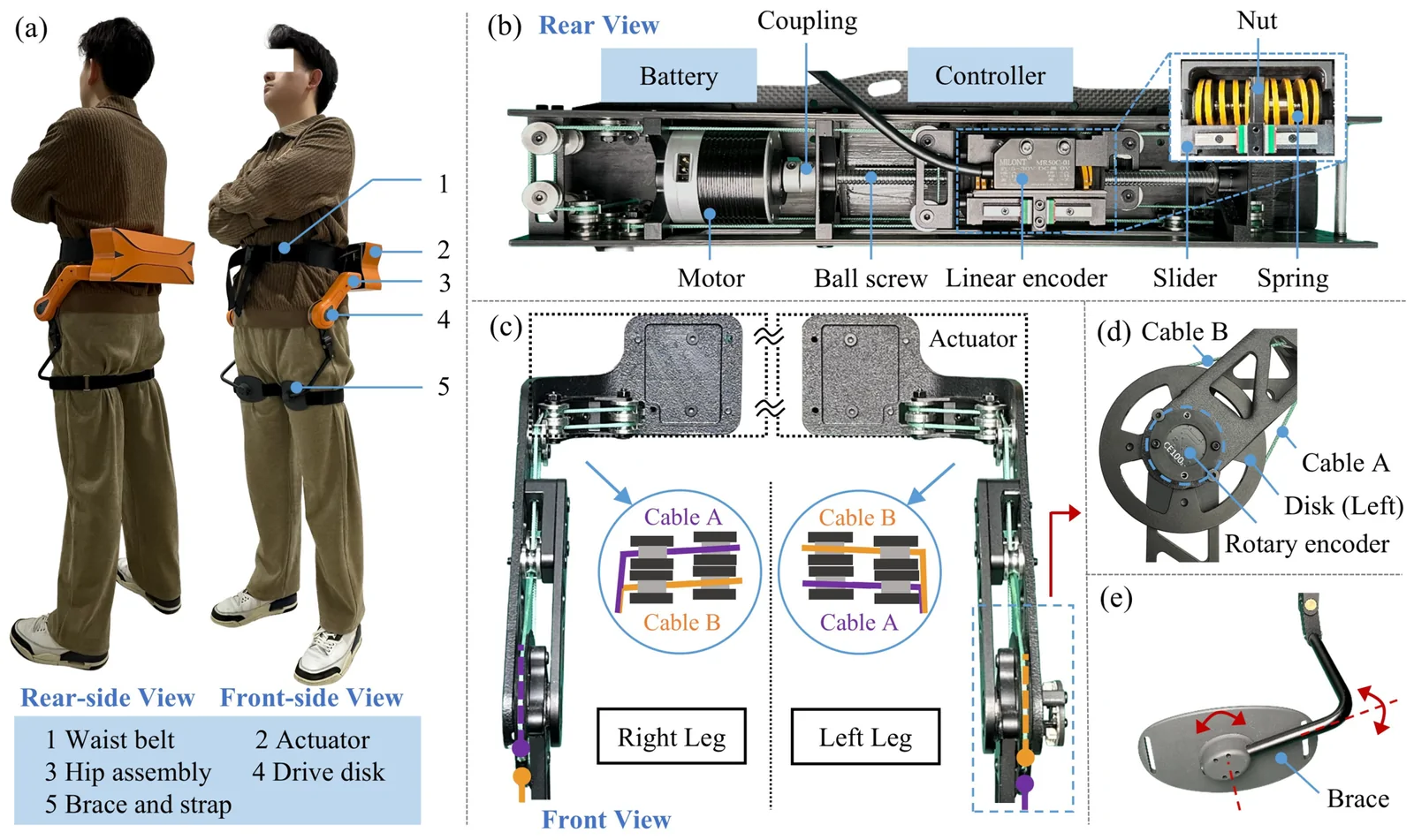
(**a**) Overall structure and schematic illustration of the hip exoskeleton (front-side and rear-side views). (**b**) Structure of the actuator. (**c**) Hip assemblies and external cable system. (**d**) Structure of the drive disk and location of the rotary encoder. (**e**) Two degrees of freedom in the lower limb brace.

The overall structure of the hip exoskeleton prototype is shown in Figure 3a. The actuator serves as the main body of the exoskeleton and is mounted on a waist belt via adjustable spacer plates, allowing the belt height to be tuned to accommodate users of different body sizes. The lower part of the actuator integrates the drive motor and the SEA, while the upper part houses the battery and controller. The left and right hip assemblies are symmetrically attached to the two sides of the actuator; when worn, the hip-drive disks (70 mm in diameter) are aligned with the anatomical hip-joint centers. The lower limb braces are positioned on the anterior side of the user’s thighs, secured using straps. The primary structural components of the system are fabricated from 6061 aluminum alloy and carbon fiber to ensure sufficient structural strength, while the outer casing is manufactured from plastic using 3D printing. The nominal maximum assistive torque on a single side of the prototype is 8.75 N·m (determined by current springs with a stiffness of 57.6 N/mm and a maximum spring force of 500 N). The total weight of the exoskeleton prototype is 3.2 kg.

Figure 3b presents a detailed view of the components of the actuator. The lower section comprises the SEA assembly, in which a motor (RMD-H-50-15, Micro Actuator Technology Co., Ltd., power 100W, Suzhou, China) drives a ball screw (pitch 2 mm) via a coupling, converting rotary motion into the linear translation of the nut. The nut compresses a set of springs and drives a slider to generate cable tension. The spring deflection is measured by a magnetic linear encoder (MR50C, Milont Technology Co., Ltd., accuracy 5 μm, Shenzhen, China). The upper section accommodates the battery on the left side and the controller (Raspberry Pi 5, 8 GB RAM, Raspberry Pi Ltd., Cambridge, UK) on the right side.

Figure 3c mainly shows the hip assemblies and the external cable system. The external cable system includes two cables that extend from the actuator, with the ends of the two cables connected to different sides of two drive disks. This way, when driving, they provide opposite assistance either side sides to help with walking. As shown in Figure 3d, the hip-joint motion is measured by a rotary encoder (HT-CE100, Haitai Electromechanical Equipment Co., Ltd., Suzhou, China) mounted on the outside of the drive disk. Figure 3e shows the two degrees of freedom in the lower limb brace to ensure wearing comfort.

## 3. Exoskeleton Control

### 3.1. Modeling and Control of SEA

To directly deliver the desired interaction force to the user, force control is adopted is adopted in the exoskeleton system rather than position control [11]. Based on the linear dynamic model of the SEA, an anti-disturbance force controller for human assistance is developed, which consists of a feedforward term and a PD-type feedback term.

**Figure 4:**
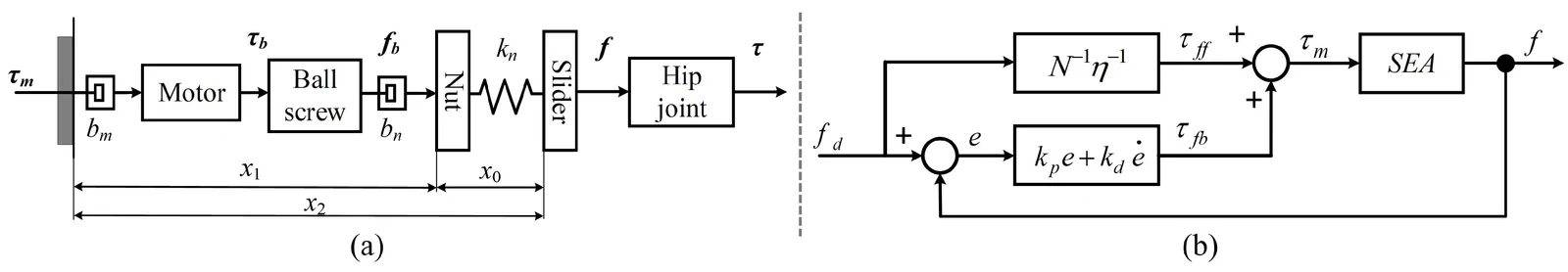
(**a**) SEA model. (**b**) SEA controller.

In our previous work, the modeling (Figure 4a) and analysis of the SEA were completed, and the corresponding transfer function of the SEA was derived [16,17], which can be expressed as follows:

(1) $$P_{\tau }\left(s\right)=\frac{f(s)}{\tau_{m}\left(s\right)}=\frac{N\eta k_{n}}{ms^{2}+bs+k_{n}}$$

In Equation (1), $m=m_{h}+N^{2}\eta \left(J_{m}+J_{n}\right)$ and $b=b_{h}+N^{2}\eta b_{m}$. τ denotes the total assistive torque applied to both hips, and $\tau_{m}$ represents the motor’s output torque. The parameter *N* is the transmission ratio of the ball screw, and $k_{n}$ is the spring stiffness, which can be identified through prior calibration. $J_{m}$ and $J_{n}$ are the rotational inertias of the motor and the ball screw, respectively. $m_{n}$ is the mass of the nut, $b_{n}$ is the damping associated with the nut’s translational motion, and $b_{m}$ is the equivalent damping on the motor side.

The output force (*f*) of the SEA is transmitted to the left and right hip joints. Assuming identical transmission parameters and equal force distribution on both sides, the assistive torque applied to a single hip joint ($\tau_{s}$) is one-half of the total assistive torque (τ). The corresponding torque relationship is expressed as follows:

(2) $$\tau_{s}=\frac{\tau }{2}=\frac{f}{2}r$$

where *r* is the effective radius of the hip-joint drive disk. All torque values reported in the subsequent experimental results refer to the single-side hip-joint torque ($\tau_{s}$).

As shown in Figure 4b, the motor command generated by the controller consists of a feedforward term and a feedback term, which can be expressed as follows:

(3) $$\tau_{m}=\tau_{\mathrm{ff}}+\tau_{\mathrm{fb}}$$

The feedforward term is designed to scale the desired torque at the approximate output of the SEA so as to minimize the magnitude of the feedback term [18]. According to the SEA transfer function, the feedforward term can be expressed as follows:

(4) $$\tau_{\mathrm{ff}}=N^{-1}\eta^{-1}f_{d}$$

where $f_{d}$ is the force command of the SEA. The feedback term compensates for unmodeled friction effects that are not captured in the SEA dynamics [19] and is formulated as follows:

(5) $$\tau_{\mathrm{fb}}=k_{p}e+k_{d}\dot{e};\;e=f_{d}-f$$

where *e* denotes the error between the commanded force and the measured force. $k_{p}$ and $k_{d}$ are the proportional and derivative gains, respectively. The controller gains were tuned using the PID Tuner toolbox in MATLAB R2023a during experimental validation.

### 3.2. Walking Assistance Strategy

In our previous work, an assistance strategy based on an underactuated mechanism was proposed for ankle-joint motion [20]. Given that the hip and ankle joints exhibit similar periodic kinematic characteristics during walking [2,15], the present study builds upon the underactuated control strategy developed in our earlier work to validate the feasibility of the underactuated hip exoskeleton structure proposed in this work.

Although the hip and ankle joints exhibit similar periodic characteristics during walking, differences in joint torque generation patterns [2,15], as well as in exoskeleton structural design, lead to several distinctions between the assistance strategy adopted in this study and that proposed in our previous work [20].

**Figure 5:**
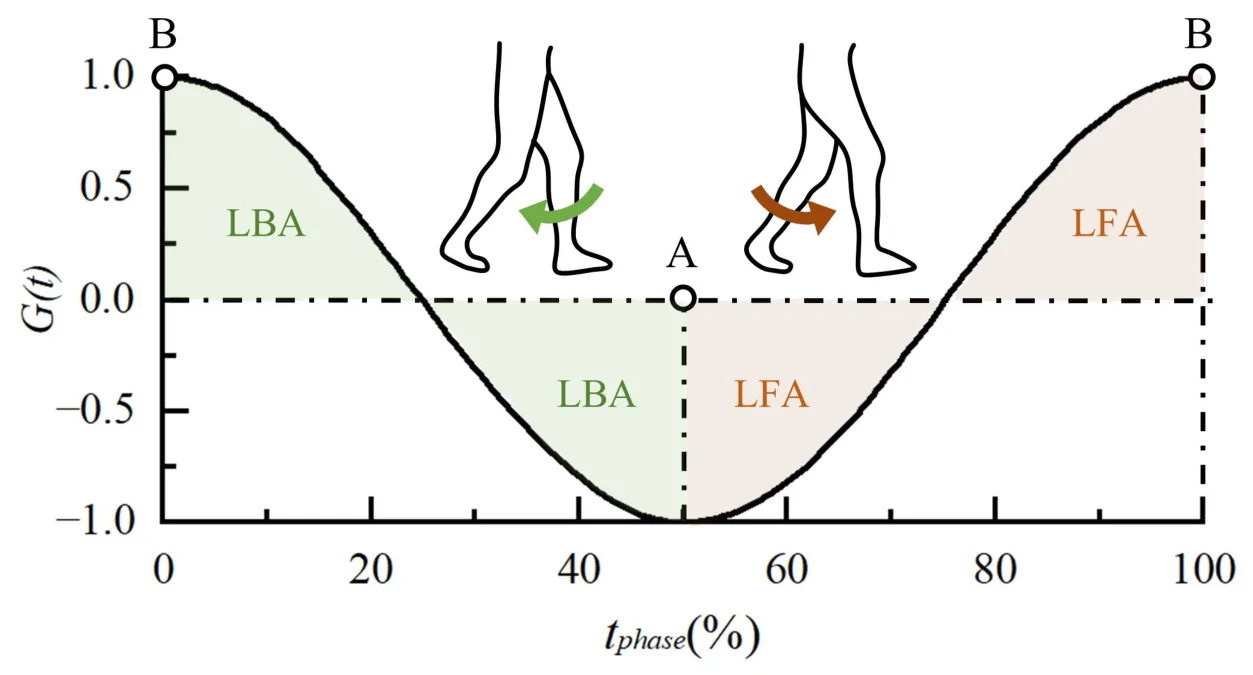
Profile of the normalized assistive function G(t) over one gait cycle. The gait cycle starts at the positive peak of the left hip-joint angle. The positive direction of G(t) is defined as opposite to the actual hip-joint torque. The meanings of A and B correspond to the letters in Figure 1b.

1. Difference in the gait recognition part:
  In this work, gait recognition is based on the angular measurement obtained from a rotary encoder mounted on one side of the drive disk. To reduce system cost while ensuring reliable detection, a single rotary encoder is employed for angle acquisition (installed outside the left disk). The local maximum of the positive angular peak is denoted as α and this moment is defined as the start of a gait cycle. This angular maximum α is continuously updated during walking to ensure robust and accurate gait-cycle identification.
2. Difference in the assistive strategy part:
  After the gait recognition phase, the walking assistance phase is initiated, during which the magnitude of the assistive force generated by the SEA is determined by the following function:
  (6) f=A·G(t)
  where *A* denotes the amplitude of the force output by the SEA and G(t) is a normalized force-scaling function matched to the human hip-joint torque profile. The approximately sinusoidal characteristic of the hip-joint torque profile during walking shown in Figure 1b is extracted to construct an assistive function suitable for the proposed exoskeleton structure. According to the description in Section 2.1, the assistance is divided into two stages (LFA and LBA). As shown in Figure 5, G(t) is defined as a sinusoidal function of the following form:
  (7) $$G\left(t\right)=-\sin\left(2\pi \left(t_{\mathrm{phase}}-t_{0}\right)\right)$$
  where $t_{\mathrm{phase}}$ denotes the normalized time representing the proportion of the current instant within an assistance cycle and $t_{0}$ is a phase-delay constant selected to match the hip-joint torque profile. As described above, the gait cycle begins when the left hip angle reaches its maximum value (α). At this instant, $t_{\mathrm{phase}}=0$, and $t_{0}$ is set to 0.25. This setting makes the assistive force reach its maximum at the beginning of the gait cycle, consistent with the strategy shown in Figure 5.

## 4. Experiments

### 4.1. Force-Tracking Performance of the SEA

**Figure 6:**
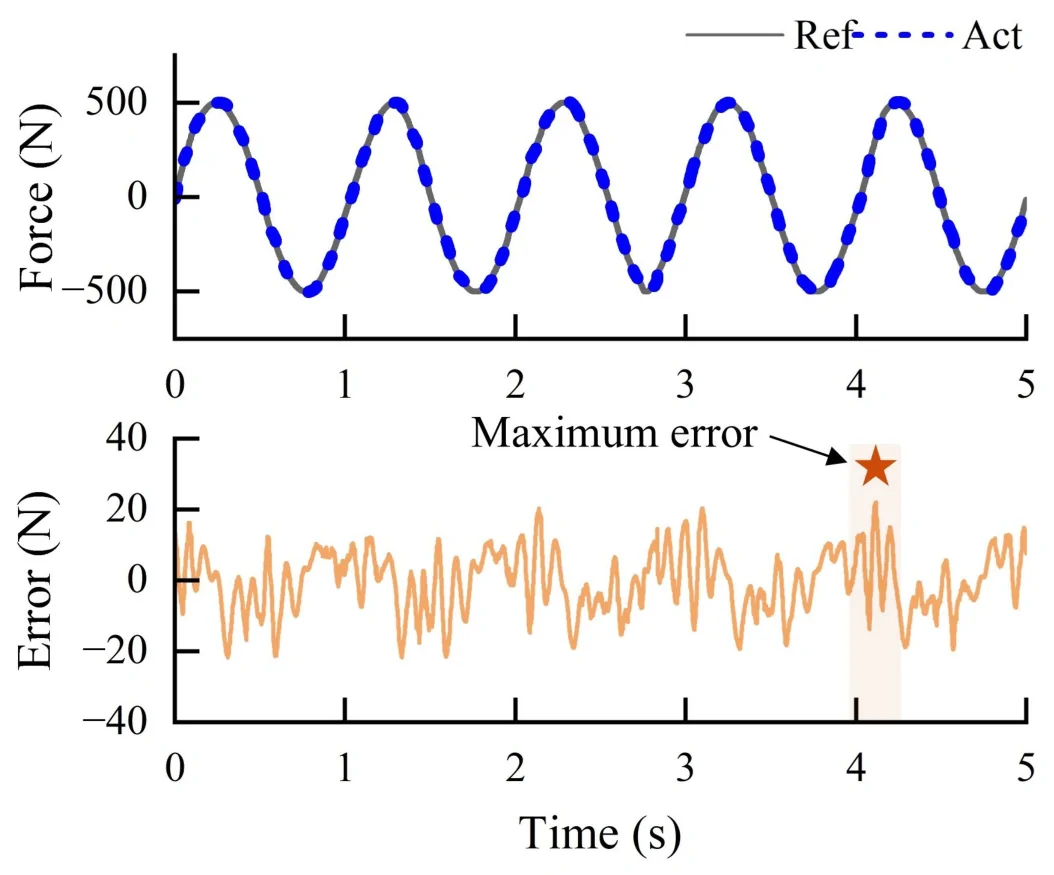
Large (1 Hz, from −500 N to 500 N) force-tracking performance of the SEA. In the first figure, the “Ref” curve represents the reference force, and “Act” represents the actual force.

To ensure accurate execution of the force-control commands, the force-tracking performance of the SEA was first evaluated. During the experiment, the SEA was commanded to track a 1 Hz sinusoidal reference signal with an amplitude of 500 N. Since bilateral assistive forces are required in hip exoskeletons to ensure reliable walking assistance, the commanded force range was set from −500N to 500N. The experimental results are shown in Figure 6; the maximum tracking error of the SEA is approximately 22 N, corresponding to a force-output accuracy of 95.6%.

### 4.2. Unassisted Walking Test

**Figure 7:**
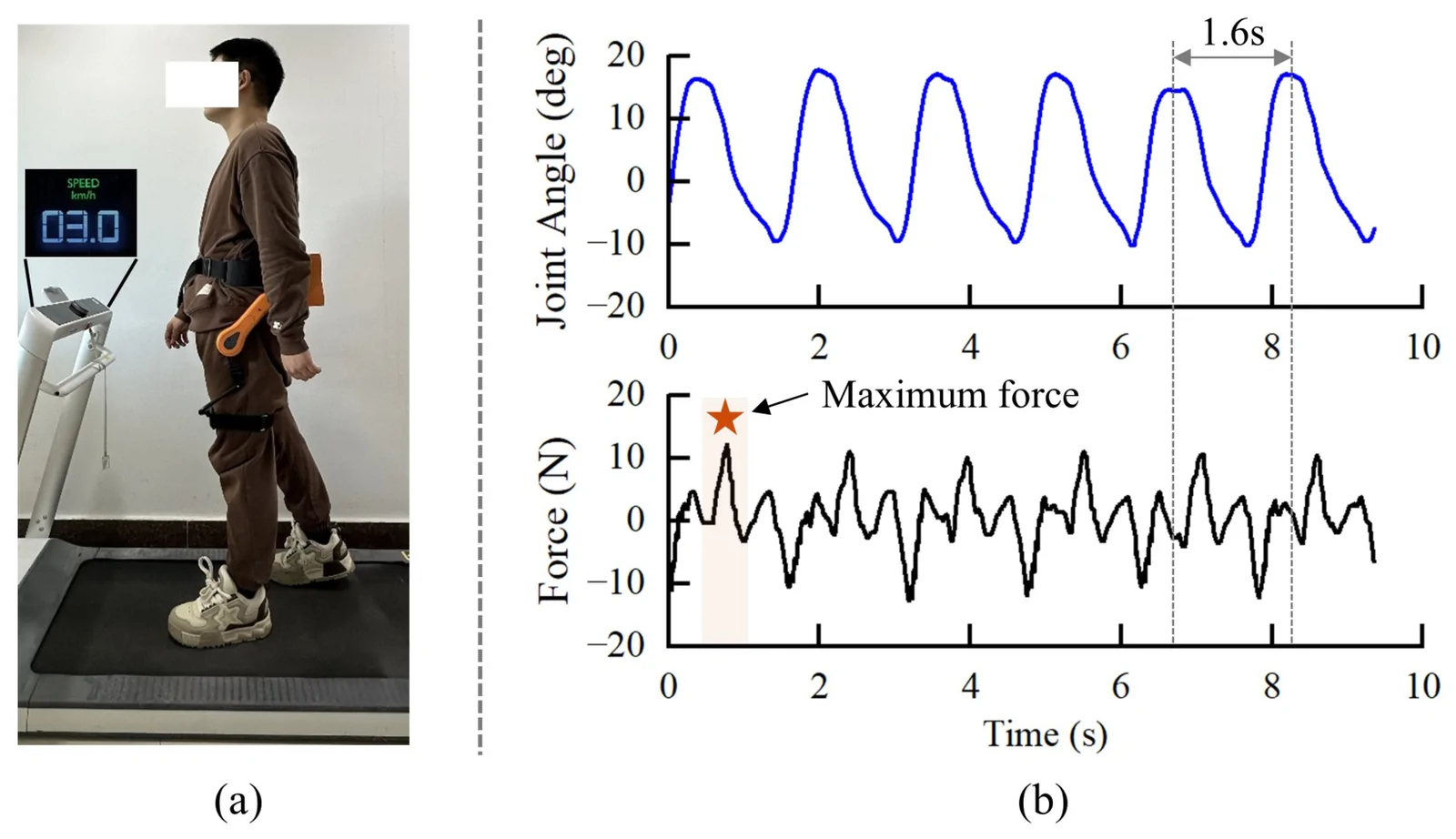
(**a**) Experimental scenario of the unassisted test. (**b**) Results of the unassisted test.

When no assistive force is required, the exoskeleton should allow the wearer to walk naturally with minimal mechanical resistance. Therefore, an unassisted walking test was conducted with the SEA output force set to zero to evaluate the resistance imposed on the user’s movement by the exoskeleton structure. In the unassisted walking test, Subject 1 (male; height: 1.78 m; weight: 84 kg) walked on a treadmill at a speed of 3 km/h (about 0.8 m/s) while wearing the hip exoskeleton, as shown in Figure 7a. During the experiment, the SEA output force was regulated to 0 N to maximally characterize and evaluate the inherent mechanical resistance of the device under the unassisted condition.

The results of the unassisted walking test are shown in Figure 7b. The angle profile represents the left hip-joint angle. As indicated by the results, the duration of a single gait cycle is approximately 1.6 s, and the maximum resistance generated by the device during the walking experiment is about 12 N. By considering the dimensions of the hip exoskeleton joint drive disk (70 mm in diameter), it can be further calculated that the maximum resistive torque experienced at the hip joint during unassisted walking is approximately 0.21 N·m. These experimental results demonstrate that the exoskeleton exhibits low structural resistance in the unassisted mode and does not impose a significant impediment to the user’s normal walking.

### 4.3. Assisted Walking Test

To evaluate the assistive performance of the hip exoskeleton, multiple walking tests were conducted. Three subjects were recruited for the tests: Subject 1, Subject 2 (male; height: 1.88 m; weight: 86 kg), and Subject 3 (male; height: 1.86 m; weight: 74 kg). All subjects were in good health, with no known musculoskeletal or motor impairments. During the experiments, the exoskeleton provided assistance over the entire gait cycle following the completion of the gait recognition phase, in accordance with the assistance strategy described in the Walking Assistance Strategy section. Two walking speeds—2.9 km/h (about 0.8 m/s) and 3.6 km/h (about 1.0 m/s)—were selected to simulate normal human walking conditions [21,22]. Based on the results of multiple assistance tests, the target output force of the SEA was ultimately set to 300 N to achieve an improved user experience.

**Figure 8:**
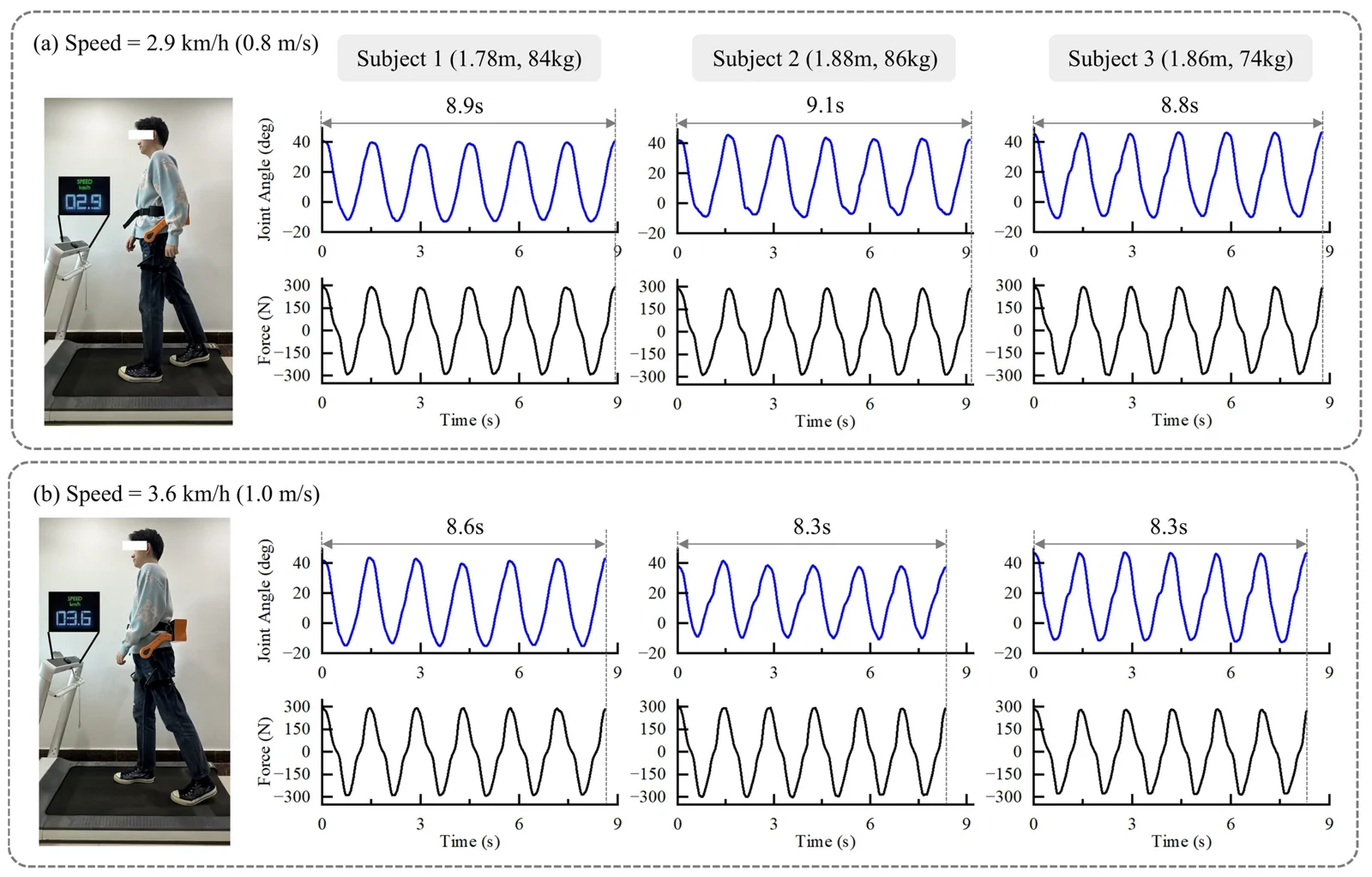
(**a**) Assistance test scenario and results at 2.9 km/h. (**b**) Assistance test scenario and results at 3.6 km/h. A total of three subjects were recruited for the experiment, and the figure shows the experimental scenario for Subject 2.

Figure 8a shows the experimental results of three subjects walking at a speed of 0.8 m/s. Under this condition, the actual maximum feedback force (*f*) of the SEA was 291.29 N, 288.54 N, and 289.46 N, corresponding to force output accuracies of 97.10%, 96.18%, and 96.49%, respectively. The maximum assistive hip torques were 5.10 N·m, 5.05 N·m, and 5.07 N·m. Figure 8b presents the experimental results at a walking speed of 1.0 m/s. In this case, the actual maximum feedback force (*f*) of the SEA was 291.29 N, 294.04 N, and 283.96 N, with corresponding force output accuracies of 97.10%, 98.01%, and 94.65%, respectively. The resulting maximum assistive hip torques were 5.10 N·m, 5.15 N·m, and 4.97 N·m. It should be noted that the force feedback is closely related to the subjects’ gait patterns, walking speeds, and gait cycles. Overall, the experimental results demonstrate that the proposed system achieves high force-control accuracy across different participants and walking speeds.

### 4.4. Free Sitting Movement Test

In Section 2.2, the principle of the differential cable structure is described in detail, and it is highlighted that this structure allows users to perform free sitting movement. To validate the feasibility of the differential cable structure, we conducted a free sitting-movement test in the absence of assistive force.

**Figure 9:**
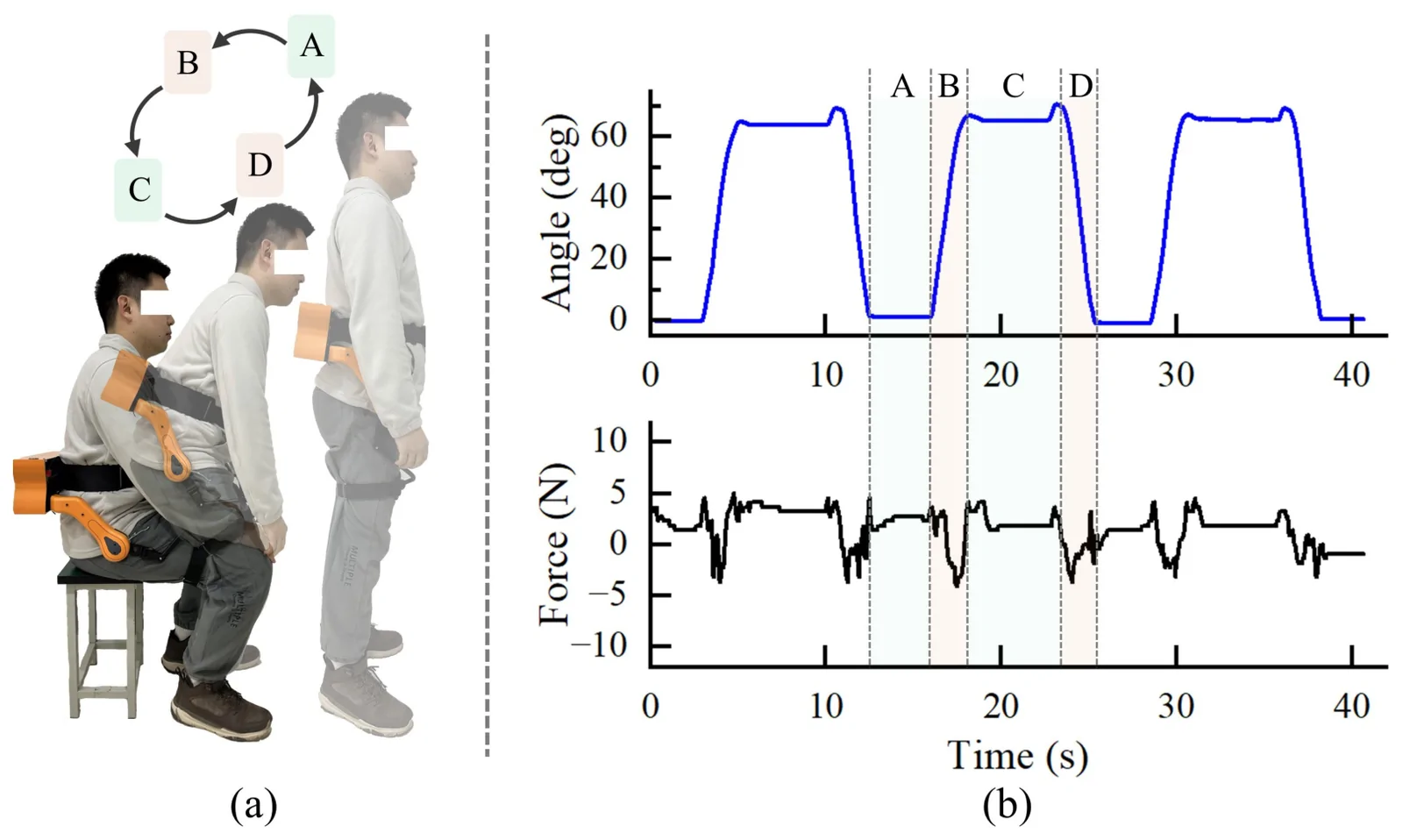
(**a**) Free sitting-movement test setup. (**b**) Free sitting-movement test results. A–D represents a complete sit–stand action.

During the experiment, the SEA output force was initially set to 0 N, placing the exoskeleton in an unassisted state. The participant then donned the exoskeleton normally and was instructed to perform three consecutive sit-to-stand cycles. The experimental procedure is illustrated in Figure 9a.

Figure 9b presents the results of the free sitting-movement test. A–D represents a complete sit–stand action, where A and C represent the static states of standing and sitting, respectively, and B and D represent the processes of sitting down and standing up, respectively. From the joint angle trajectories, it can be observed that the participant’s hip angle varied approximately between 0° and 70° during the stand-to-sit movements. The hip angle was measured using an encoder mounted on the drive-disk side of the hip joint. The force profiles represent the resistive forces encountered throughout the stand-to-sit movements. The peak resistive force was approximately 5 N, which is considerably lower than the resistance experienced during unassisted walking.

### 4.5. Muscle Activation Test

To evaluate the effectiveness of the proposed hip exoskeleton, a series of experiments was conducted to simulate normal walking conditions. Surface electromyography (EMG) signals were measured to assess muscle activation.

EMG signals were collected using gForceOct Wireless EMG sensors (OYMotion Technology Co., Ltd., Shanghai, China) with a sampling frequency of 1000 Hz. EMG signals were recorded from four muscles that play important roles in hip flexion and extension during walking: the iliopsoas (IP), rectus femoris (RF), gluteus maximus (GM), and biceps femoris (BF) of the hamstring group [23]. The experimental setup was identical to that used in the previous unassisted walking test. The subject walked on a treadmill at a speed of 3 km/h under two conditions: without wearing the exoskeleton and wearing the exoskeleton. At the beginning of each trial, the subject was instructed to walk with a natural gait. Data acquisition started after the EMG signals stabilized, and each recording lasted approximately 30 s (20 gait cycles). To reduce the influence of random variability, the above procedure was repeated five times under both experimental conditions.

The collected EMG data were processed using the following procedure (data processing method adapted from [24]): First, the EMG signals were filtered and rectified using a band-pass filter (20–450 Hz, fourth-order zero-phase Butterworth) and a band-stop filter (58–62 Hz, fourth-order zero-phase Butterworth). Then, the signals were envelope-processed using a low-pass filter (7–15 Hz). For visualization, the time-series data were normalized over the gait cycle to 101 data points. The mean of the peak values across all envelope-processed gait cycles under the condition without the exoskeleton was used as the reference for peak normalization. The existing data were then normalized against this reference to obtain both the average and peak muscle activation levels.

**Figure 10:**
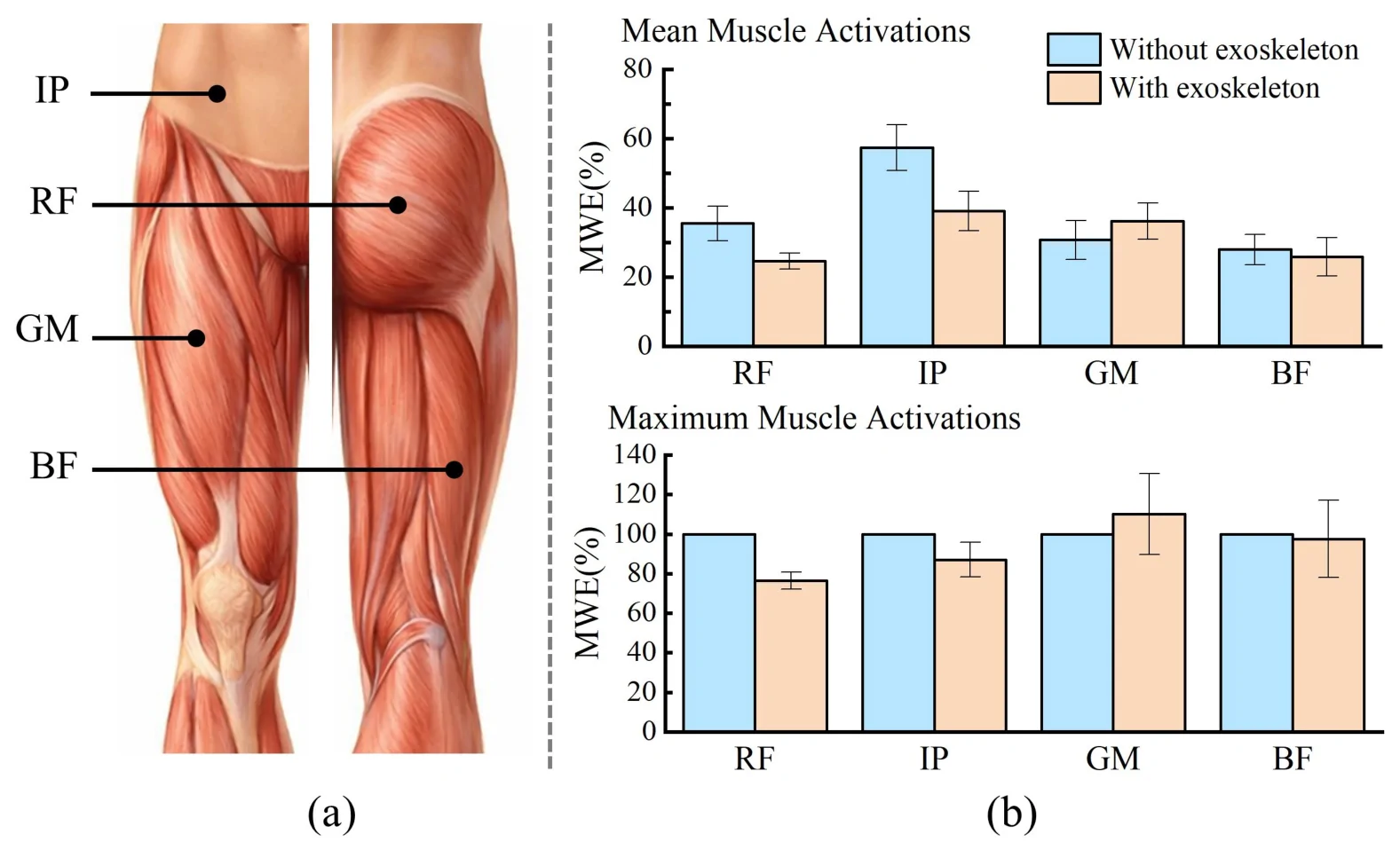
(**a**) Schematic of muscle locations for muscle activation test. (**b**) Muscle activation results. Error bars represent the standard deviation, and MWE represents maximum muscle activations without the exoskeleton.

The muscle activation test results are shown in Figure 10b. The normalized mean activations of the RF, IP, and BF muscles decreased to varying degrees when wearing the exoskeleton. The IP exhibited the largest reduction of 18.24%. The RF and BF muscles also showed reductions of 10.93% and 2.11%, respectively. In contrast, the GM showed a slight increase in mean activation, with a rise of 5.37%. The peak activation of the RF, IP, and BF muscles decreased noticeably when wearing the exoskeleton. The RF exhibited the largest reduction of 23.62%. The IP and BF muscles also showed reductions of 12.92% and 2.42%, respectively. In contrast, the GM showed increased activation when assisted by the exoskeleton, with a rise of 10.23%.

## 5. Discussion

In the division of the gait cycle, we categorized the cycle into two stages—LFA and LBA—as explained in Section 2.1, applying opposing directions of assistance in each phase to facilitate natural walking. However, as shown in Figure 1b, the angular changes of the hip joints on both sides are not strictly monotonic throughout each phase (for instance, during the LFA phase, the left hip angle initially decreases before rising significantly). This occurs because the angles of both hip joints exhibit a decreasing trend during the interval between the hip reaching its maximum angle and the foot on that side making contact with the ground. However, within a single stage, the torque directions of every hip joint remains the same, so the non-monotonicity of the angles does not affect the effectiveness of the current assistance strategy.

The proposed exoskeleton employs an underactuated dual-cable design, enabling free sitting movement while reducing the number of actuators and the device’s weight. During walking assistance, the actuator delivers approximately equal-amplitude reverse assist to the left and right hip joints via dual cables. Consequently, this structure is better suited for a symmetric assistance strategy involving anti-phase motion between the left and right sides. Incorporating the sinusoidal characteristics of the hip-joint motion profile (Figure 1b), a sine-based symmetric assistance strategy effectively matches the structural characteristics of the system. Relevant studies also indicate that a sinusoidal assistance strategy based on gait phases can be effectively applied to walking assistance with lower-limb exoskeletons [25,26]. To achieve a more precise assistance strategy, we plan to improve the system’s adaptability through structural optimization in the future, such as by adding variable stiffness devices or decoupling mechanisms. On top of that, we could combine more accurate gait recognition with personalized assistance strategies to provide more precise assistance.

In the unassisted walking test, the actual feedback force could not be maintained at zero (Figure 7b), primarily due to the system’s passive impedance. Since the feedback force is derived from the spring deformation measured by a linear encoder, the movement of the hip joint during walking drives the slider in the opposite direction via the cable. This causes slight tensile or compressive deformation of the spring, thereby generating a residual force output. The redistribution of cable tension during the reversal of the hip-joint movement induces additional local force peaks; meanwhile, cable friction, slider resistance, and encoder zero-point errors may also introduce certain deviations. The figure shows that the peak feedback force is approximately 12 N—far lower than the peak active assistance force of 300 N—indicating that the exoskeleton minimally interferes with normal walking when unassisted and exhibits good transparency. It should also be noted that the hip angle during walking is not strictly symmetric and opposite, showing that the proposed differential cable structure allows hip motion to deviate from an ideal symmetric and opposite pattern, thereby helping accommodate natural variations in hip motion during walking.

In the assisted walking tests, the walking assistance curve effectively tracked the preset standard sinusoidal assistance pattern, although slight deviations remained in the local waveform (Figure 8). This is because the human gait cycle inherently involves natural fluctuations, although it is approximately sinusoidal in nature. Hip-joint movement alters the loading state of the spring, making it difficult for the actual assistive force to maintain a perfectly standard sinusoidal profile. It was observed that the hip-angle amplitude under the assisted walking test was larger than that under the unassisted walking test. This may be attributed to the inertial effect during the transition between assistance stages, which caused a transient continuation of hip motion and consequently increased the angular amplitude.

During assisted walking at different speeds (Figure 8), Subject 1’s gait cycle showed no significant changes, whereas the gait cycles of Subjects 2 and 3 varied more noticeably with speed. This suggests that Subject 1 primarily adapts to changes in walking speed by adjusting step length or stride length rather than significantly altering cadence. Consequently, his gait cycle remains relatively stable across different speeds.

In the free sitting-movement test, as shown in Figure 9, subjects are able to smoothly complete the entire sitting–standing process at a normal speed (approximately 2 s for a single sitting or standing motion) [27]. The hip-joint angle varies roughly between 0° and 70°, depending on the wearer’s physique and the positioning of the exoskeleton. In this process, the slider should theoretically not undergo displacement. However, large-angle changes at the hip joint cause a redistribution of cable length and tension. Slight slippage and localized friction of the cable along the transmission path induce minute relative displacements between the slider and the linear encoder readhead. Consequently, the feedback force curve still exhibits minor fluctuations.

In the muscle activation test, the activation levels of the GM are higher when wearing the exoskeleton than in the unassisted condition. This may be attributed to the additional load introduced by the exoskeleton’s actuator, which increases the mechanical work of the hip extensor muscles (e.g., GM) during walking, thereby leading to higher muscle activation levels [28]. In addition, the human hip-joint torque profile is not an ideal sinusoid (Figure 1b). The optimal timing of assistance may also vary among individuals. Therefore, a standard sinusoidal assistance profile cannot fully match the optimal assistance phase for every participant. This mismatch may cause a slight increase in the activity of some muscles. We can improve the assistive performance of exoskeletons by optimizing the battery and structure to reduce exoskeleton weight in the future work. Meanwhile, we will develop an adaptive assistance strategy that adjusts the timing and magnitude of assistance based on real-time information regarding gait and muscle fatigue.

The main limitation of this work can be summarized as follows. The small size of the drive disk limits the maximum assistive torque, primarily due to the constraints of the screw travel. In future work, a pulley system could be employed to increase the effective travel, allowing a larger drive disk to generate higher assistive torque.

## 6. Conclusions

This paper proposes an underactuated hip exoskeleton that achieves system lightweighting by reducing the number of motors, providing periodic walking assistance while enabling free sitting movement. Bilateral hip-joint assistance is provided through differential cable drive achieved by two independent cables. Walking and free sitting-movement tests validate the feasibility of the differential cable structure in accommodating non-strictly symmetric hip motion and enabling free sitting movement. In addition, an SEA enables accurate force control. Experimental results show that, under a target assistive force of 300 N, the system achieves a maximum force-tracking accuracy of 98.01% in walking tests, corresponding to a peak assistive torque of 5.15 N·m. Muscle activation tests indicate that the exoskeleton effectively reduces muscle activation during walking, with mean activation reduced by up to 18.24% and peak activation reduced by up to 23.62%.

## Data Availability

The data presented in this study are available upon request from the corresponding author.
